# Identification of a new *Cordyceps javanica* fungus isolate and its toxicity evaluation against Asian citrus psyllid

**DOI:** 10.1002/mbo3.760

**Published:** 2018-11-13

**Authors:** Da Ou, Li‐He Zhang, Chang‐Fei Guo, Xiao‐Sheng Chen, Shaukat Ali, Bao‐Li Qiu

**Affiliations:** ^1^ Key Laboratory of Bio‐Pesticide Innovation and Application South China Agricultural University Guangzhou China; ^2^ College of Forestry and Landscape Architecture South China Agricultural University Guangzhou China; ^3^ Engineering Research Center of Biological Control Ministry of Education Guangzhou China

**Keywords:** Asian citrus psyllid, biological control, *Cordyceps javanica*, entomopathogenic fungi, isolation

## Abstract

The Asian citrus psyllid, *Diaphorina citri* Kuwayama, is the most serious pest of citrus worldwide. It acts as a vector for a group of phloem‐limited bacteria (*Candidatus* Liberibacter spp.) that causes Huanglongbing (HLB) disease. Thus, *D. citri* management is an important strategy against HLB, and biological control is currently considered as the most effective method because of the unsustainable and negative side effects of chemical control. Here, we isolated a new strain of entomopathogenic fungus, *Cordyceps javanica* (GZQ‐1), from one cadaver of *D. citri* adult based on its morphological and phylogenetic data. Five conidial concentrations of the *C. javanica* pathogen (1 × 10^3^, 1 × 10^4^, 1 × 10^5^, 1 × 10^6^, and 1 × 10^7^ conidia/ml) were assessed against six life stages of *D. citri* (1st‐5th instar nymphs and adults). Results showed that *C. javanica* GZQ‐1 was highly pathogenic to *D. citri* nymphs (69.49%–90.87% mortality) and adults (69.98% mortality). The LC_50_ and LT_50_ values of *C. javanica* against 1st‐2nd instar (younger), 3rd‐4th instar (middle aged), 5th instar (older), and adults were 1.20 × 10^5^, 1.10 × 10^6^, 4.47 × 10^6^, 8.12 × 10^6^ conidia/ml and 4.25, 4.51, 5.17, 5.49 days, respectively. Moreover, glasshouse experiments indicated that this *C. javanica* GZQ‐1 caused higher infection rates of *D. citri* adults compared to two other fungal strains we previously isolated in the laboratory, *Cordyceps fumosorosea* (IF010) and *Metarhizium anisopliae* (CNGD7).

## 1 INTRODUCTION

The Asian citrus psyllid, *Diaphorina citri* Kuwayama (Hemiptera: Liviidae), is one of the most economically important pests of citrus worldwide. It serves as a vector of phloem‐limited bacteria (*Candidatus *Liberibacter spp.), which can cause Huanglongbing (HLB; citrus greening disease), a devastating disease of citrus (Bové, 2006; Lin, 1956). Thus, population management of *D. citri* is a basic and most important strategy in blocking the spread of HLB disease. Intensive chemical control of *D. citri* is the primary strategy currently advocated for HLB management. However, this strategy is pernicious and unsustainable with several negative side effects such as chemical residues, resistance, and pest resurgence being noted (Tansey, Vanaclocha, Monzo, Jones, & Stansly, 2017).

In view of the undesirable side effects of pesticides, biological control has become an advantageous development direction for the prevention and control of agricultural pests. Within this area, entomopathogenic fungi play a particularly important role. In addition to efficacy and cost, there are several advantages of using entomopathogenic fungi: they have broad‐spectrum insecticidal activity, diversified species range, complex metabolic types, and offer appropriate safety levels for humans and other non‐target organisms (Lacey, Frutos, Kaya, & Vail, 2001). They are also easy to mass‐produce and development of host resistance against them is unlikely to occur. They also infect their insect hosts in a unique way entering the hemocoel cavity through the external cuticle, where they absorb nutrients, produce immunosuppressive toxins (Vilcinskas & Götz, 1999), damage the host cells, and interact with the gut microbiota to promote host death. They finally kill the host, and the given fungus grows out the cuticle of the dead insect releasing conidiophores to infect other host individuals (Wang & Wang, 2017; Wei et al., 2017; Xiong et al., 2013). All this promotes their good application potential within invertebrate pest control strategies.

Different natural enemies of *D. citri* including parasitoids, predators, and entomopathogenic fungi have been reported and applied in practice to manage this pest. Among the parasitoids, *Tamarixia radiate* (Waterston) and *Diaphorencyrtus aligarhensis* (Shafee, Alam and Argarwal) have been utilized to control *D. citri* population in Mauritius, Reunion Island, and the United States (Chen & Stansly, 2014; Chen, Triana, & Stansly, 2017). The predatory natural enemies of *D. citri* investigated includes ladybirds, predatory mites, lacewings, spiders, ants, and preying mantis (Juan‐Blasco, Qureshi, Urbaneja, & Stansly, 2012; Michaud, Olsen, & Olsen, 2004; Navarrete, Mcauslane, Deyrup, & Peña, 2013).

Entomopathogenic fungi account for more than 60% of insect pathogenic microorganisms. The utilization of entomopathogenic fungi to control *D. citri* offers a great development opportunity on account of their significant epidemic potential and the convenience of production. Lezama‐Gutiérrez et al. (2012) determined the virulence of three entomopathogenic fungi against *D. citri* in the field and found 42%, 50%, and 22% mortality of *D. citri* adults when they infected with *Beauveria bassiana*, *Metarhizium anisopliae,* and *Cordyceps fumosorosea* (Wize) Kepler, B. Shrestha & Spatafora, comb. nov. (MycoBank MB820980), formerly *Isaria fumosorosea*， respectively. Ibarra‐Cortes et al. (2018) ascertained the fatality rate of *C. fumosorosea*, *B. bassiana*, and *M. anisopliae* to be 28%, 55%, and 43%, respectively, to *D. citri* adults, and 47%, 69%, and 100%, respectively, to the nymphs when applying 1 × 10^7^ conidia/ml suspensions of the individual fungi under laboratory conditions.

In the present study, a new strain of entomopathogenic fungus, *Cordyceps javanica* (Friederichs & Bally), formerly *Isaria javanica* (Friederichs & Bally; Kepler et al., 2017), isolated from an adult cadaver of *D. citri* was identified through morphological as well as phylogenetic data. The pathogenicity of this newly isolated strain to *D. citri* was analyzed through bioassays against different developmental stages.

## 2 MATERIALS AND METHODS

### 2.1 Psyllid cadaver collection and fungus isolation

A new fresh entomopathogenic fungal strain was isolated from an adult *D. citri* cadaver collected from a glasshouse at South China Agricultural University (SCAU) in Guangzhou, China. The carcass was infiltrated in water containing 5% Tween‐80 and shaken vigorously to re‐suspend the fungal spores or the mycelium fragments present on the cuticle surface. Then 10 μl of this suspension was plated on standard PDA medium. After 1–2 days of incubation at 27°C, individual germinations (or mycelium regeneration) were transferred to a new plate. Thereafter, this isolate was submitted to several rounds of purification in order to follow morphological stability after the successive transfers. This purified strain was named as “GZQ‐1,” and deposited in Guangdong Microbial Culture Collection Center (GDMCC) with the deposition number GDMCC 60437.

Before morphological observation, this GZQ‐1 isolate was firstly cultivated on SDAY/4 medium (SDAY/4:10 g/L dextrose, 2.5 g/L peptone, 2.5 g/L yeast extract, and 15 g/L agar) in Petri dishes placed within a biochemical incubator (26 ± 1°C, 60% RH, L:D = 14:10). Fungal conidia were then harvested from plates after 5 days, suspended in 0.05% Tween 80 (v/v) and shaken on a vortex mixer for 10 min. The conidial suspension was then filtered, counted and adjusted to the target concentration for pathogenicity tests. The infected nymphs and adults of *D. citri* were observed and recorded under a binocular microscope (ZEISS, Discovery.V20, Germany), and preliminary morphology was identified based on phenotypic properties and pathogenic fungi morphology (Jaber, Mercier, Knio, Brun, & Kambris, 2016).

### 2.2 Morphological observation

For the morphometric evaluation of the GZQ‐1 isolate, its micro‐cultures were first grown on SDAY/4 and incubated at 27°C for 10 days. Slides were then prepared with lactophenol/blue cotton (10:1) and examined with phase contrast optics under an optical microscope Olympus BX51 (Microscopy GmbH, Gottingen, Germany). Images of the conidia were photographed digitally with an Axio Cam HRc camera (Carl Zeiss) using the Axion Vision SE64 Release 4.9.1 software.

To measure the growth rate and conidia yield of GZQ‐1, the fungi were cultured on SDAY/4 at 27°C for 10 days, and then the conidia were scraped from the plates and suspended in 10 ml of sterile water. Following this, the suspension was filtered through Miracloth held in a funnel and quantified using a hemocytometer. The growth rate of GZQ‐1 hypha was measured based on their morphology on SDAY/4 medium plate on day 10 of culturing. Both the examinations were repeated three times.

### 2.3 DNA extraction and ITS sequencing of the GZQ‐1 isolate

Total DNA of the GZQ‐1 isolate was isolated from each sample of the test strains using a fungal DNA kit following the standard manufacturer's instructions (Fungal DNA Kit; Sangon Biotech, Shanghai, China). Purified DNA specimens were amplified with universal primers, ITS4: 5’‐TCCTCCGCTTATTGATATGC‐3’, and ITS5: 5’‐GGAAGTAAAAGTCGTAACAAGG‐3’. Each PCR was carried out in 25 μl volumes, containing 12.5 μl 2 × HiFiTaq PCR StarMix buffer (GenStar, China), 1 μl of each primer, 1 μl of total DNA (53.1 ng/μl), and 9.5 μl of ddH_2_O. The reaction was then performed using the following reaction cycles: initial denaturation at 94°C for 3 min, followed by 35 cycles of denaturation at 94°C for 30 s, annealing at 55°C for 30 s and extension at 72°C for 45 s, then a final extension phase at 72°C for 10 min. PCR products were visualized by 1.0% agarose gel electrophoresis, stained with Gold View in 0.5 × TBE buffer (Sangon, Shanghai, China) and photographed under UV light. Following this the target PCR products were sent to The Beijing Genomics Institute (BGI; Shenzhen, China) for complete bidirectional sequencing with PCR primers.

The resulting sequences were checked and aligned using Lasergene v7.1 (DNASTAR, Inc., Madison, WI). Then the similarity of sequences compared with homologous sequences deposited in GenBank (Table 1) was calculated using “BLAST” tools on the National Center for Biotechnology Information (NCBI) website, and a resulting phylogenetic tree constructed using MEGA 6 software (Felsenstein, 1985; Saitou & Nei, 1987). *C. javanica* strain CBS 134.22 and *C. fumosorosea* strain CBS 107.10 were used as the Ex‐type strains. *Metarhizium anisopliae* strains ZJ and YD2‐1‐8 were used as the out group in the phylogeny analysis.

**Table 1:** The reference entomopathogenic fungi from GenBank used in phylogenetic analysis. (“*” is the GenBank accession number of GZQ‐1; “N/A” unlabeled in NCBI)

Species	GenBank number	Strain no.	Host	Location
*Cordyceps * *javanica*	MG742216*	GZQ‐1	*Diaphorina citri*	Guangzhou, China
*Cordyceps * *javanica*	AY624186	Ex‐type CBS 134.22	*Hypothenemus hampei*	Java
*Cordyceps * *javanica*	MG837718	ACP	*Diaphorina citri*	Fuzhou, China
*Cordyceps * *javanica*	KM234218	CHE‐CNRCB 307/7	*Bemisia tabaci*	Armeria, Mexico
*Cordyceps * *javanica*	KM234213	CHE‐CNRCB 303/2	*Bemisia tabaci*	Armeria, Mexico
*Cordyceps * *javanica*	KM234212	CHE‐CNRCB 303	*Bemisia tabaci*	Armeria, Mexico
*Cordyceps * *javanica*	KT225592	CHE‐CNRCB 310	*Spodoptera littoralis*	France
*Cordyceps * *fumosorosea*	AY624182	CBS 244.31	Butter	Ireland
*Cordyceps * *fumosorosea*	AY624183	CBS 375.70	Food	Japan
*Cordyceps * *fumosorosea*	AY624184	Ex‐type CBS 107.10	N/A	France
*Beauveria bassiana*	KX093961	YT‐11	*Bombyx mori*	Shandong, China
*Beauveria bassiana*	KF772868	GA‐1	*Micromelalopha troglodyta*	Hubei, China
*Beauveria bassiana*	KF772861	YD‐1	*Dendroctonus punctatus*	Hubei, China
*Beauveria brongniartii*	HQ380853	ART376	*Melolontha melolontha*	Innertkirchen, Switzerland
*Beauveria brongniartii*	JX110381	SASR HHB32B	*Galleria mellonella*	South Africa
*Beauveria brongniartii*	JF947191	ARSEF8153	*Agrilus planipennis*	Ontario, Canada
*Metarhizium anisopliae*	JN377427	ZJ	*Rhynchophorus ferrugineus*	Hainan，China
*Metarhizium anisopliae*	KX380792	YD2‐1‐8	Soil	Nanchang, China

Phylogenetic hypotheses were analyzed independently for ITS with Maximum Likelihood (ML) using MEGA 6 software based on the Tamura‐Nei model for ITS, and a discrete gamma distribution was applied for each analysis with 1,000 bootstrap replicates (ML BS). The resulting trees are visualized in Figure 4.

### 2.4 Plants and insects in testing

*Murraya paniculata* (L) Jacks plants were used in this study. New seedling plants were cultured in 30‐cm‐diameter plastic pots containing a soil‐sand mixture (10% sand, 5% clay, and 85% peat) in a glasshouse at ambient temperature and photoperiod.

The colony of *D. citri* was first collected from *M. paniculata* plants at SCAU and then continuously reared on *M. paniculata* seedlings in a glasshouse onsite at SCAU at 26–28°C and 60%–65% relative humidity under a 14:10 hr (L:D) photoperiod. New plants with fresh shoots were provided for the *D. citri* culture once a month. For bioassay analysis, six life stages of *D. citri* were used: 1st–5th nymphal instars and mature adults. Nymphs were collected into Petri dishes using a fine camel hairbrush, and following morphological examination under a stereomicroscope, were classified into their individual nymphal stages based on morphological features.

### 2.5 Pathogenicity testing of the GZQ‐1 isolate

#### 2.5.1 Laboratory bioassays

Experiments were performed in an artificial climate incubator (26 ± 1°C, 90% RH and photoperiod 14:10 hr (L:D)). Conidial suspensions of GZQ‐1 were diluted with sterile distilled water to five concentrations (1 × 10^3^, 1 × 10^4^, 1 × 10^5^, 1 × 10^6^, and 1 × 10^7^ conidia/ml). The LC_50_ values and LT_50_ values of this GZQ‐1 isolate on *D. citri* younger nymphs (1st–2nd instar), middle and older nymphs (3rd–5th instar), and adults (3 days after emergence) were measured. Double sterile water was used as a negative control. Different instars of *D. citri* were sprayed with each concentration of conidial suspension as well as the ddH_2_O control (till the leaf surface was covered with beads of solution). Approximately 30–40 individuals of different instar *D. citri* nymphs or adults were examined in each bioassay, with each assay being replicated three times.

#### 2.5.2 Glasshouse bioassays

The glasshouse bioassay of GZQ‐1 isolate to *D. citri* adults (3 days following emergence) were carried out in the autumn of 2017 at SCAU (average temperature 24–30°C, 50%–60% RH). *C. fumosorosea* (IF010) and *M. anisopliae* (CNGD7), which we have isolated in SCAU (Dai et al., 2017; Freed, Jin, & Ren, 2011) were also tested as positive controls. The conidial suspensions of these fungi (including GZQ‐1) were diluted with sterile distilled water to 1 × 10^7^ conidia/ml. Approximately 30–40 insects were sprayed with the individual conidial suspensions or ddH_2_O as control in each bioassay, with each assay replicated three times.

The infected *D. citri* by the three entomopathogenic fungi were observed daily, and the dead individuals were removed and underwent further incubation to promote fungal growth. The average mortalities of *D. citri* caused by the tested fungi were calculated. The mathematical calculation of lethal concentrations LC_50_ and confidence limits were carried out by the method of probit analysis (Zhang et al., 2018).

## 3 RESULTS

### 3.1 Morphological identification of GZQ‐1 isolate

**Figure 1:**
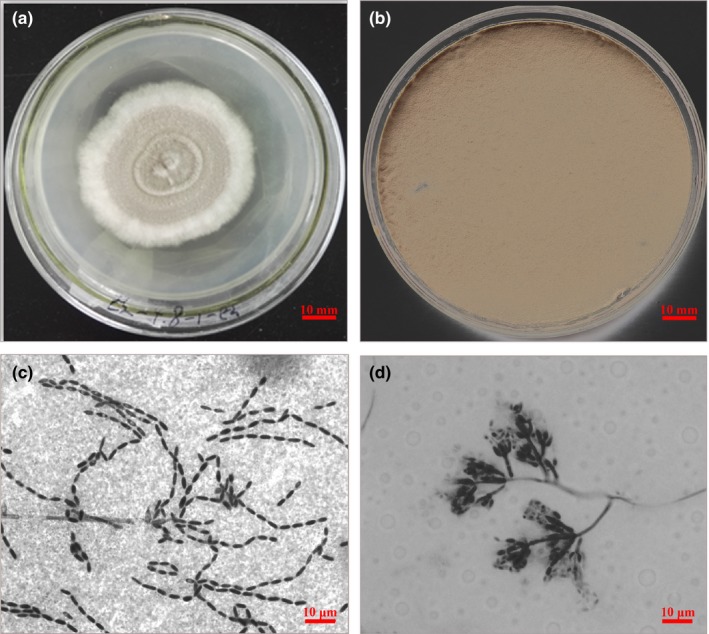
The morphology of *Cordyceps javanica* GZQ‐1 isolate on SDAY/4. (a) The upper side of *C. javanica* colony on SDAY/4 on 4th day; (b) The upper side of *C. javanica* mature colony on SDAY/4 on 14th day; (c) *C. javanica* GZQ‐1: chains of conidia. (d) *C. javanica* GZQ‐1: Phialides with developing conidia

The morphological features of GZQ‐1 isolate are illustrated in Figure 1. The fungal colonies had a concentric ring pattern. Mycelium texture was velvet like. The center of the colony was pale pink while spores on the edge of the colony were white in color with radial growth (Figure 1a). The mature colony was brownish gray (Figure 1b). The conidiophores were straight with a long ovoid conidial shape linked into a chain, and the phialides were characterized by a wide globose basal portion with a long distal neck (Figure 1c,d). The morphological identification and infection observation revealed that GZQ‐1 isolate was *Cordyceps javanica.*

**Figure 2:**
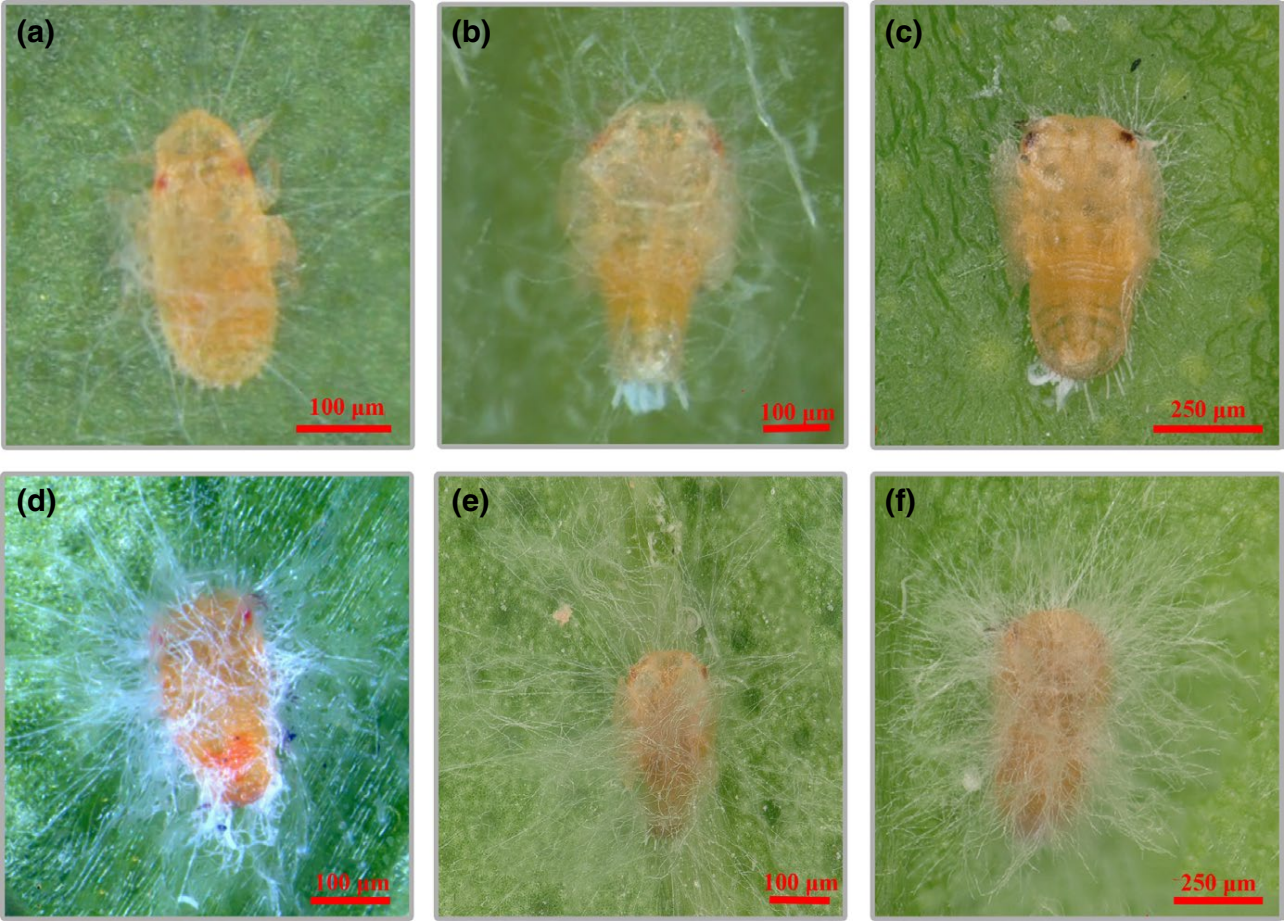
The infection phenotype of *Diaphorina citri* nymphs treated with *Cordyceps javanica* (1 × 10^7^ conidia/ml). Panels a, b, and c were the 1st, 2nd, and 3rd nymphs of *D. citri* on 2nd day after infection while panels d, e, and f were the 1st, 2nd, and 3rd nymphs on the 7th day after infection

**Figure 3:**
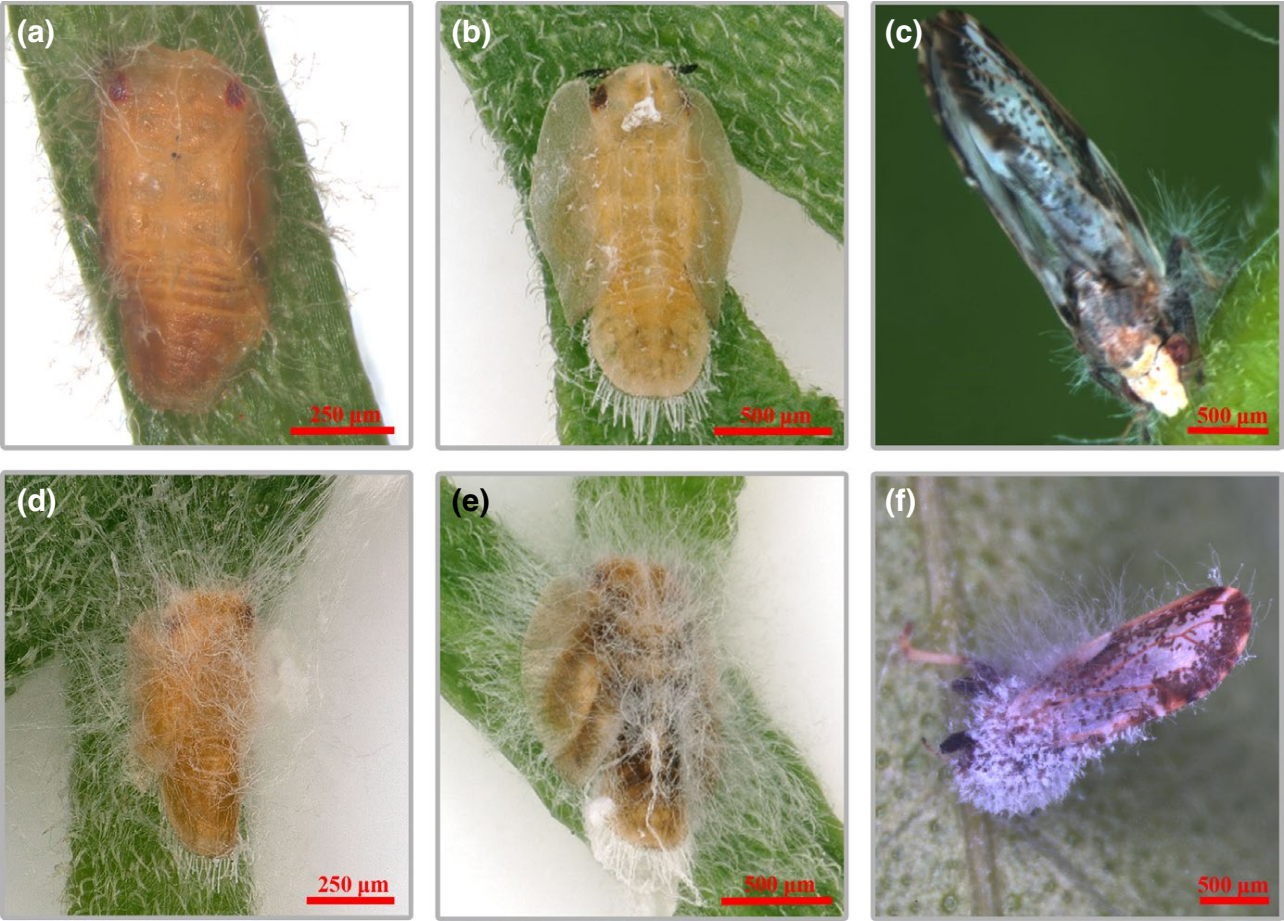
The infection phenotype of *Diaphorina citri* older nymphs and adults treated with *Cordyceps javanica* (1 × 10^7^ conidia/ml). Panels a, b, and c were the 4th and5th nymphs and mature adults of *D. citri* on 2nd day after infection while panels d, e, and f were the 4th and 5th nymphs and mature adults on the 7th day after infection

When *D. citri* nymphs and adults were infected by the GZQ‐1 isolate in the glasshouse, the insects were observed to move slowly and suffer twitching of legs and antennae after 48 hr infection. Infected adult *D. citri* scratched actively, clung tightly to branches and leaves until they were completely covered with hyphae and finally died. Microscopic observation showed that fungal hyphae emerging from the tarsi and intersegmental regions of the legs of infected *D. citri* after 48–72 hr had grown from the metamere and intersegmental membranes. Then covering the whole of the *D. citri* including the wings, it caused death after seven days or longer (Figures 2 and 3).

### 3.2 Sequencing of the ITS genes and phylogenetic analysis

**Figure 4:**
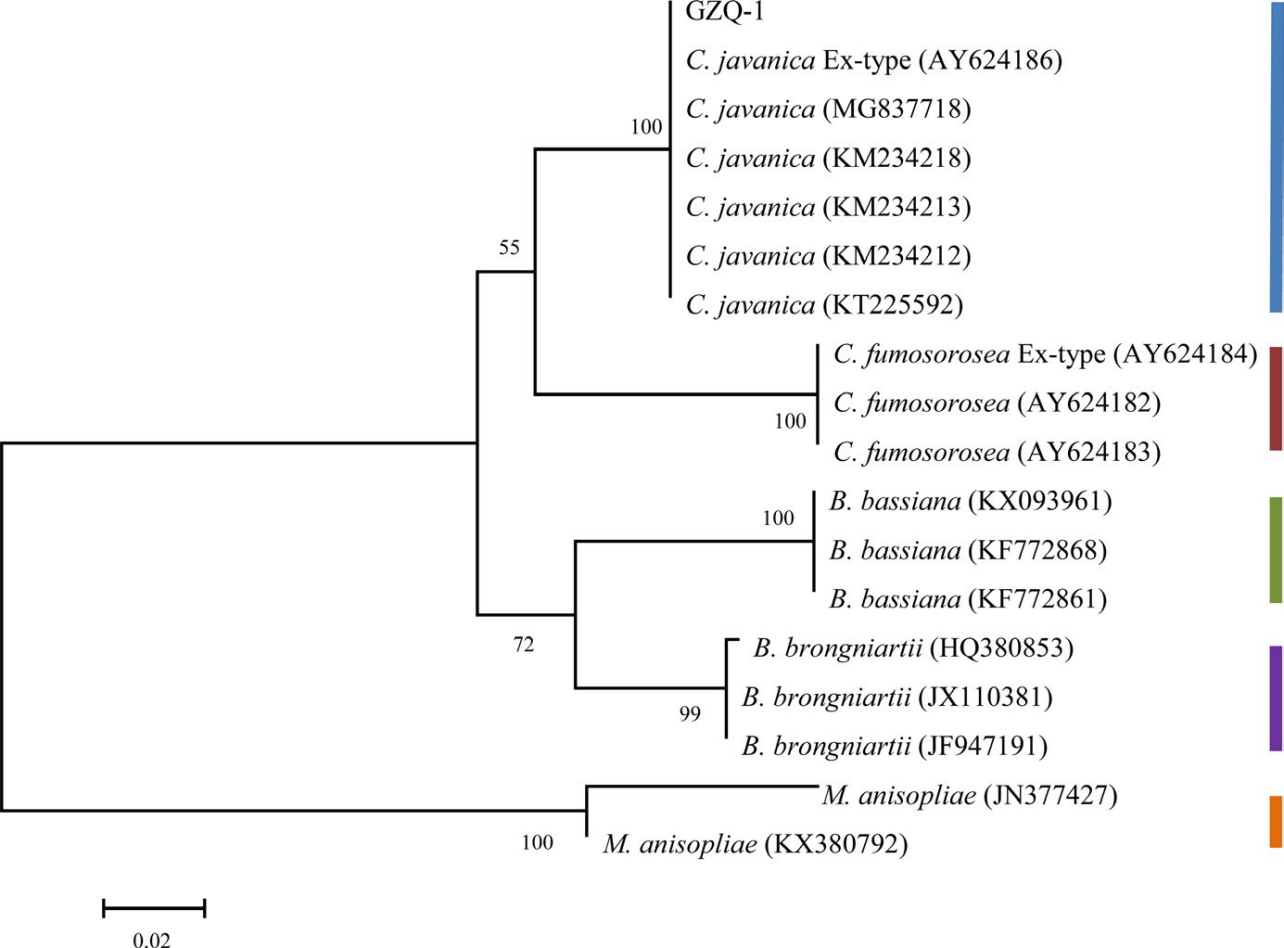
Majority rule consensus phylogram from the Bayesian analysis based on the sequences of the internal transcribed spacer (ITS) region for 18 isolates (GZQ‐1 isolate, *Cordyceps javanica, Cordyceps fumosorosea, Beauveria brongniartii, Beauveria bassiana*. *Metarhizium anisopliae* was used as the outgroup). Support values are shown for ML BS. The bars in different color show the different fungal species but different strains clustered into one branch in the phylogenetic tree

Polymerase chain reaction amplification and DNA sequencing results indicated that the rDNA‐ITS gene of GZQ‐1 isolate was 616 bp (data not shown), following this the DNA sequence was submitted to GenBank; where it gain the GenBank accession number of MG742216. BLAST results in GenBank revealed that the ITS gene of GZQ‐1 was 100% homologous to the *C. javanica* strain (GenBank accession number MG837718). Moreover, phylogenetic analysis showed that GZQ‐1 isolate was closely clustered in the *C. javanica* clade (Figure 4), which supported our morphological identification that the GZQ‐1 isolate is a *C. javanica* strain.

### 3.3 Pathogenicity testing of the GZQ‐1 isolate

#### 3.3.1 Laboratory bioassays

**Figure 5:**
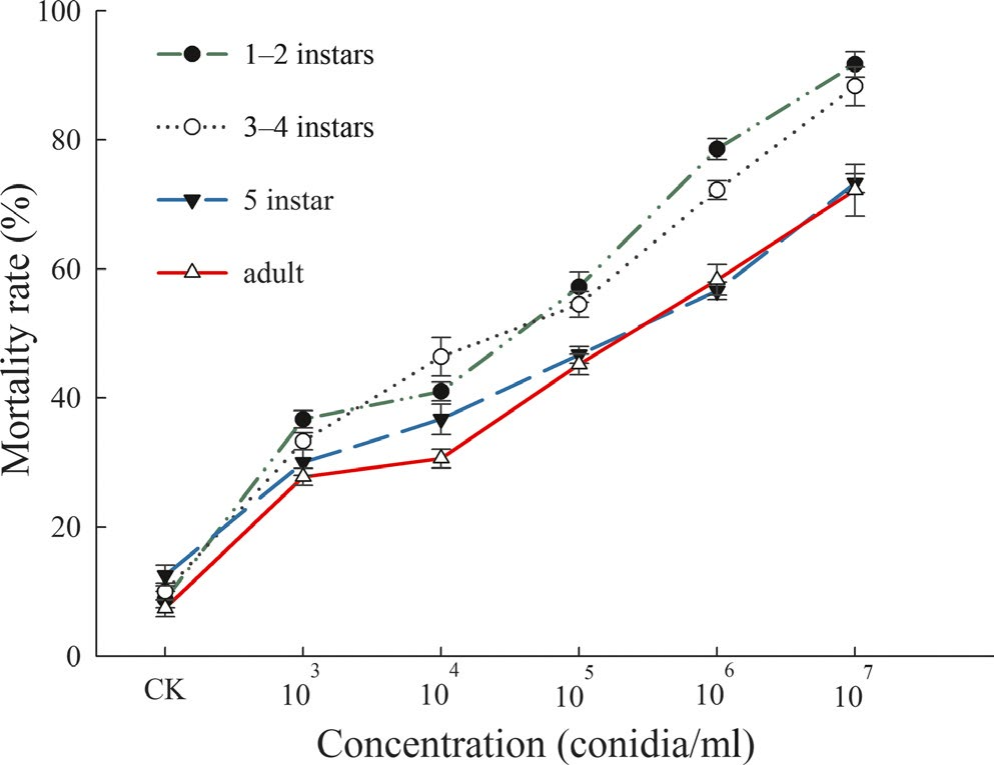
The mortality of *Diaphorina citri *different developmental stages treated with various concentrations of *Cordyceps javanica* (1 × 10^3^, ×10^4^, ×10^5^, ×10^6^, and ×10^7^ conidia/ml). Data are mean ± *SEM* of three tests

After 10 days of incubation, the diameter of *C. javanica* colony reached 42.3 ± 1.86 mm and sporulation was 2.26 × 10^8^ spores/g on SDAY/4 medium. The bioassay results showed that GZQ‐1 isolate has high pathogenicity to both the nymphs and adults of *D. citri*, with corrected mortality rates of 91.7% ± 2.36%, 88.3% ± 4.33%, 73.3% ± 3.32%, and 72.2% ± 2.94% to the 1st–2nd instar, 3rd‐4th instar, 5th instar nymphs, and adults of ACP following the 7th day of infection, respectively (Figure 5); there were significant differences among the mortality rates of 1st–2nd instar nymphs, 3rd–4th instar nymphs, 5th instar nymphs, and adult *D. citri* (*p* < 0.05).

Bioassay results showed that the LC_50_ values for *C. javanica* GZQ‐1 isolate acting on the different developmental stages of *D. citri* after 7 days were 5.19 × 10^5^, 1.27 × 10^6^, 4.47 × 10^6^, and 8.12 × 10^6^ conidia/ml, respectively (Table 2). While the LT_50_ values of GZQ‐1 isolate on different developmental stages of *D. citri* were 4.25, 4.51, 5.17, and 5.49 days, respectively (Table 3). Lethal concentration and death time increased with an increase in developmental stage of *D. citri* when sprayed with the same concentration of *C. javanica* GZQ‐1. This demonstrated that our *C. javanica* GZQ‐1 isolate was most effective against younger instar nymphs of *D. citri*.

**Table 2:** Pathogenicity regression equations for LC_50_ values of *Cordyceps javanica* against different developmental stages of *Diaphorina citri* after 7 days of infection (1 × 10^7^ conidia/ml)

Insect stages	Regression virulence model	LC_50%_ and 95% CI (conidia/ml)	*R* ^2^
1st−2nd instar nymph	*Y* = 0.129*X* − 0.085	5.19 (−13.2 ~ 19.55) × 10^5^	0.634
3rd−4th instar nymph	*Y* = 0.132*X* − 0.164	1.27 (−1.02 ~ 3.07) × 10^6^	0.724
5th instar nymph	*Y* = 0.145*X* − 0.44	4.47 (1.98 ~ 9.35) × 10^6^	0.947
Adult	*Y* = 0.436*X* − 2.07	8.12 (1.81 ~ 2.62) × 10^6^	0.961

**Table 3:** Pathogenicity regression equations for LT_50_ values of *Cordyceps javanica* against different developmental stages of *Diaphorina citri* after 7 days of infection (1 × 10^7^ conidia/ml)

Insect stages	Regression virulence model	LT_50%_ and 95% CI (d)	*R* ^2^
1st−2nd instar nymph	*Y* = 0.549*X* − 2.335	4.25 (3.87 ~ 4.64)	0.950
3rd−4th instar nymph	*Y* = 0.466*X* − 2.101	4.51 (4.08 ~ 4.96)	0.976
5th instar nymph	*Y* = 0.449*X* − 2.322	5.17 (4.73 ~ 5.70)	0.981
Adult	*Y* = 0.486*X* − 2.668	5.49 (5.09 ~ 5.96)	0.943

#### 3.3.2 Glasshouse bioassays

**Figure 6:**
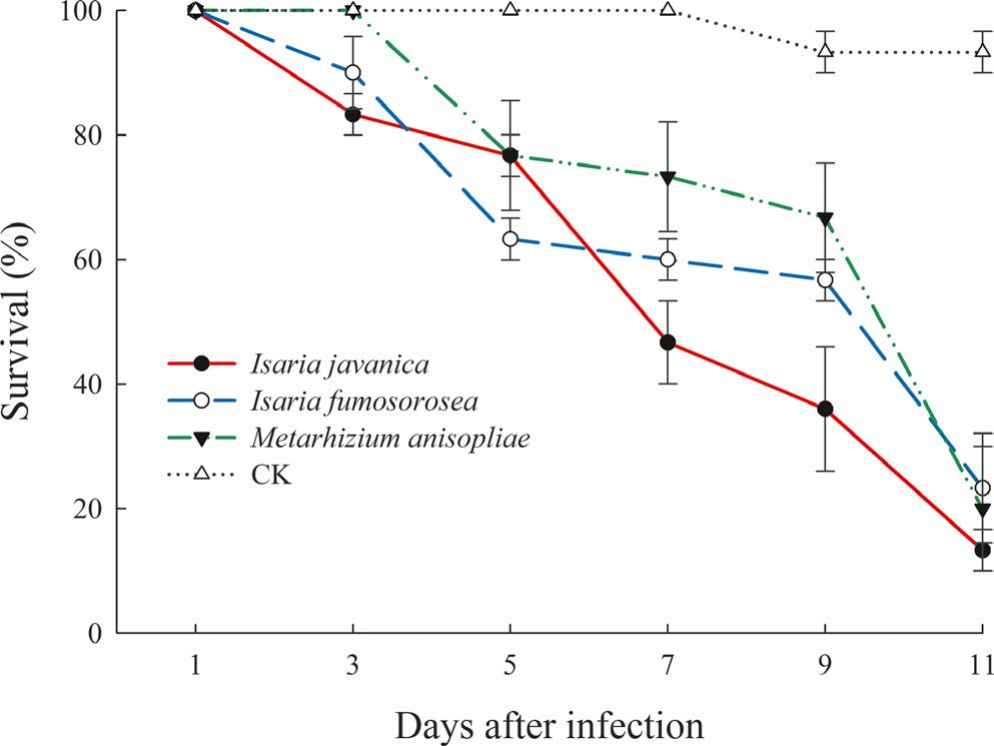
The survival rates of *Diaphorina citri* adults when infected with *Cordyceps javanica* GZQ‐1 isolate as well as *Cordyceps fumosorosea* and *Metarhizium anisopliae* (all are 1 × 10^7^ conidia/ml) under glasshouse conditions. Data are mean ± *SEM* of three tests

The semi‐field pathogenicity of GZQ‐1 isolate to *D. citri* was compared with two other excellent entomopathogenic fungi, which have been screened under laboratory conditions: *C. fumosorosea* and *M. anisopliae* at 1 × 10^7^ conidia/ml. Results showed that, when infected with *C. javanica* GZQ‐1 isolate, the survival rates of *D. citri* adults was 82.6% on the 3rd day, reducing to 35.7% on the 9th day and finally 18.3% on the 11th day. When infected with *C. fumosorosea* and *M. anisopliae*, their survival rates were 90.0% and 100% on the 3rd day, reducing to 60.0% and 66.7% on the 9th day and finally 22.3% and 20.0% on the 11th day, respectively. In contrast, only 7.7% mortality was recorded in the non‐infected controls on the 9th day (Figure 6). Results revealed that the infection and pathogenicity of *C. javanica* isolate to *D. citri* in semi‐field conditions was faster or higher than the two other fungal strains we previously isolated in the laboratory (*C. fumosorosea* (IF010) and *M. anisopliae* (CNGD7)) after 7 days of infection in the current study*.*

## 4 DISCUSSION

Numerous insect pathogenic fungi including *Paecilomyces varioti*, *C. fumosorosea*, *C. javanica*, *Beauveria. bassiana*, *M. anisopliae*, *Hirsutella citriformis*, *Acrostalagmus aphidium*, *Fusarium culmorum*, *Lecanicillium lecanii*, *Cladosporium oxysporum*, *Capnodium citri*, and *Stemphylium* sp. have been reported to control different insect pests, but only a few studies have reported the pathogenicity of *C. javanica* against *D. citri* (Faria & Wraight, 2007; Gandarilla‐Pacheco, Galán‐Wong, López‐Arroyo, Rodríguez‐Guerra, & Quintero‐zapata, 2013; Medina‐Córdova, Rosales‐Mendoza, Hernández‐Montiel, & Angulo, 2018; Meyer, Hoy, & Boucias, 2008; Vega et al., 2008). Nguyen et al. (2017) isolated two highly virulent strains of *C. javanica* against diamondback moth *Plutella xylostella* with respective LT_50_ values of 2.52 and 2.55 days.

Based on the complete morphological and molecular characteristics described for *Isaria*, Cabanillas, Leon, Humber, Murray, and Jones (2013) correctly identified *C. javanica* from the erroneous differentiated *I. poprawskii*, which was collected from a cadaver of the sweet potato whitefly, *Bemisia tabaci* biotype B in the Lower Rio Grande Valley of Texas, USA. Following this, Mellin‐Rosas, Sanchez‐Gonzalez, Cruz‐Avalos, Montesinos‐Matias, and Arredondo‐Bernal (2016) reported that their *C. javanica* CHE‐CNRCB 303 isolate can cause 95% mortality of both *D. citri* nymphs and adults. In the current study, the *C. javanica* GZQ‐1 isolate is very similar to the *C. javanica* fungi descriptions published in regards to morphological features and phenotype of colony (Gallou et al., 2016); its conidial characteristic is also consistent to the findings of Gallou et al. (2016). Moreover, the growth speed and sporulation quantity of our *C. javanica* GZQ‐1 colony was faster than that reported by Yin, Qiong‐Bo, Guo‐Hua, and Mei‐Ying (2010) and Shimazu and Takatsuka (2010).

In terms of the pathogenicity bioassays, the LC_50_ and LT_50_ values of *C. javanica* GZQ‐1 isolate showed good biological control potential for *D. citri* management. Pinto, Filho, Almeida, and Wenzel (2012) reported the pathogenicity of *B. bassiana* (IBCB 66) to *D. citri* nymphs: the LC_50_ and LC_90_ values on the 10th day of application were 0.4 × 10^6^ and 6.7 × 10^6^ conidia/ml, respectively. Hoy, Singh, and Rogers (2010) determined the pathogenicity of *C. fumosorosea* (AsCP) to *D. citri* adults, here the LT_50_ value was 4.625 days at spore concentrations of 1 × 10^7^ conidia/ml and the LC_50_ value was 6.8 × 10^7^ conidia/ml after 8 days of fungal application. Our results indicated that our *C. javanica* GZQ‐1 isolate was similar too or even better than these high quality strains concerning their pathogenicity to *D. citri.*

Under semi‐field conditions, Dai et al. (2017) observed 62% and 65% mortality of *D. citri* when treated with 1 × 10^7^ conidia/ml of *B. bassiana* (Bb‐E) and *C. fumosorosea* (IF‐B) after 7 days of infection, respectively. Lezama‐Gutiérrez et al. (2012) determined that the fatality rate of *C. fumosorosea*, *B. bassiana*, *M. anisopliae* to *D. citri* adults were 22%, 42%, and 50%, respectively, in the field. Compared to these studies and our current findings, the different pathogenicity rates of these fungi may be due to strain distinctions, different biotic, abiotic conditions, including host species, humidity, temperature, and soil type in the various studies (Bugeme, Maniania, Knapp, & Boga, 2008; Erler & Ates, 2015; Inyang, Mccartney, & Oyejola, 2000; Kim, Oh, Yoon, & Sung, 2017; Sharififard, Mossadegh, & Vazirianzadeh, 2012).

In conclusion, the efficacious strategy against Huanglongbing disease by suppressing *D. citri* populations has been required to affiliate a multi‐tactic integrated pest management (IPM) programme (Meyer et al., 2008; Weintraub & Beanland, 2006). In the current study, we identified a high pathogenic *C. javanica *GZQ‐1 isolate based on its morphology and phylogeny and estimated its potential use in Asian citrus psyllid biological control. Our study is expected to enrich the available resource library of entomopathogenic fungi and provide an alternative preparation for inclusion within *D. citri* IPM strategies.

## CONFLICT OF INTEREST

The authors declare no conflict of interest.

## AUTHORS CONTRIBUTION

DO and BLQ designed the study. DO, LHZ, and CFG performed the experiments and analyzed the data. XSC and SA participated in data analysis and photography. DO, BLQ, and SA wrote the paper. BLQ supported the grants.

## ETHICS STATEMENT

None required.

## Data Availability

All DNA sequences are submitted to GenBank with Accession number MG742216. All data generated or analyzed during this study are included in this published article.
